# Application of the Cascade of Care Framework to Guide Evidence-Informed Implementation of Anal Cancer Screening Guidelines

**DOI:** 10.1177/10732748261466365

**Published:** 2026-07-10

**Authors:** Ann N. Burchell, Troy Grennan, Christine Fahim, Dina Gaid, Apondi J. Odhiambo, Shuk On Annie Leung

**Affiliations:** 1 Department of Family and Community Medicine, Temerty Faculty of Medicine, University of TorontoToronto, Ontario, Canada; 2 MAP Centre for Urban Health Solutions, Li Ka Shing Knowledge Institute, St. Michael’s Hospital, Unity Health Toronto, Toronto, Ontario, Canada; 3 Dalla Lana School of Public Health, University of Toronto, Toronto, Ontario, Canada; 4 Clinical Prevention Services, British Columbia Centre for Disease Control, Vancouver, British Columbia, Canada; 5 Department of Medicine, University of British Columbia, Vancouver, British Columbia, Canada; 6 Knowledge Translation Program, Li Ka Shing Knowledge Institute, St. Michael’s Hospital, Unity Health Toronto, Toronto, Ontario, Canada; 7 Division of Gynecologic Oncology, Department of Obstetrics and Gynecology, McGill University Health Centre, Montreal, Quebec, Canada

**Keywords:** cancer screening, design, health care, HPV, population

## Abstract

Most squamous cell carcinomas of the anus, or anal cancers, are attributable to persistent infection with the human papillomavirus (HPV), analogous to cervical cancer. Although rare in the general population, rates of anal cancer are high among people with immune-compromising conditions or a history of HPV-related cancers. Recent evidence demonstrates that treatment of high-grade squamous intraepithelial lesions (HSIL), a precursor of anal cancer, prevents progression to anal cancer. This has motivated the international release of several guideline statements now recommending anal cancer screening. For a new practice of cancer screening to be integrated into routine care for at-risk populations, implementation strategies will need adaptation for local settings. Our aim is to illustrate the potential value of combining the methods of implementation science with the anal screening cascade framework to support evidence-informed adoption of the guidelines across the different clinical services points where patients might undergo anal cancer screening. We point out means by which research activity could be directed, with particular attention to identifying and addressing care gaps to inform the adoption and scale-up of anal cancer screening. These approaches may help to guide local decisions regarding first steps for anal cancer screening implementation, including determining which populations may need enhanced effort, and to identify where more preparation may be needed.

## Plain Language Summary

It is time to understand how to best implement anal cancer screening. Although rare in the general population, rates of anal cancer are 20 to 100 times higher among people living with HIV, or with solid organ transplants, or among women who have had human papillomavirus-related cancers. Like cervical and colorectal cancer, early detection and treatment can prevent anal cancer. Unlike cervical or colorectal cancer, most jurisdictions do not routinely screen for anal cancer. In 2024, the first international guidelines for anal cancer screening were released. They recommend that clinicians offer screening to people at the highest risk—those who are at greater than 10 times the risk of the general population. In our paper, we describe strategies to evaluate early attempts at anal cancer screening implementation to support efforts to ensure that anal cancer screening becomes part of regular health care for people in whom it is recommended.

## Introduction

A promising new addition to the list of screen-preventable cancers is anal cancer, or more specifically squamous cell carcinoma of the anus. Most (90%+) are caused by persistent infection with oncogenic types of the sexually-transmitted human papillomavirus (HPV), which is also the cause of cervical cancer.^1^ Anal cancers develop through the malignant progression of precancers called high-grade squamous intraepithelial lesions (HSIL). Anal cancer is rare in the general population, with global estimates of 0.54 diagnoses per 100,000 in 2022, with slightly higher rates in high income than low and middle income countries.^2^ However, the risk for anal cancer is 10 to 100 times higher in certain populations with immune compromising conditions (e.g., those living with HIV, solid organ transplant recipients) or with a history of HPV-related disease at other sites.^1^ These rates are comparable to cancers with established screening programs: colorectal, breast, and cervical cancer.^3^

The similarity of the natural histories of cervical and anal cancers, combined with the success of cervical screening programs, has motivated the investigation of screening as secondary prevention of anal cancer.^4^ Ideally, one would detect and treat HSIL before it progresses to cancer. One reason why anal cancer screening was not routinely practiced is because it was only recently that robust evidence emerged regarding the efficacy of anal HSIL treatment in preventing cancer development.^4^ In 2022, the ANCHOR study, a large randomized clinical trial in the United States, revealed that HSIL treatment reduces the risk of progression to anal cancer by 57% (95% confidence interval 6 to 80%) among people living with HIV aged ≥35 years of all genders compared to surveillance (“watchful waiting”).^5^ Immediate treatment was of such benefit that the trial was stopped to offer treatment to all participants.^6^

Soon after the reporting of findings from ANCHOR, several international and country-specific organizations made recommendations for anal cancer screening for people living with HIV and, in some cases, other high-risk populations. In early 2024, the International Anal Neoplasia Society (IANS) released their consensus guidelines with recommendations on populations to screen, screening tests, and thresholds for referral to high-resolution anoscopy (HRA), an examination of the anal canal mucosa, using a colposcope which provides magnification, aimed at identifying HSIL and other abnormalities through visualization and biopsies, analogous to cervical colposcopy.^7^ The IANS guidelines now recommend screening for anal precancer via cytology and/or HPV testing. Country-specific statements followed, including from the United States, Brazil, Portugal, and France, reviewed by Albuquerque,^8^ and in Australia^9^ and Germany.^10^

Many of the guidelines are consistent in their recommendations regarding screening of men who have sex with men (MSM) living with HIV and women with a history of solid organ transplantation or vulvar cancer/precancer. There is more heterogeneity across guidelines in terms of other targeted screening populations and which screening test combinations to use.

The recommended procedures involve swabbing the anal lining for (1) cytology testing to identify cellular abnormalities in the anal squamous epithelium (“anal Pap test”), or (2) testing for oncogenic HPV types, or (3) a combination of both tests (either co-testing or reflex testing).^7^ Most guidelines also recommend assessment of symptoms and a digital anal rectal exam (DARE, to detect early-stage cancers)^11^ at the time of testing for precancer. Patients with no abnormality on DARE but who have screen-detected abnormalities on cytology to HPV testing are to proceed to diagnostic testing using HRA, with biopsy of abnormal-appearing areas.

If HSIL is confirmed with histology, the preferred treatment is ablation (although other treatments are in active investigation).^7,12^ Following treatment, patients are followed for recurrence that may require additional treatment.^13,14^

### Challenges and Opportunities for Implementation of Anal Cancer Screening

Many clinicians and health systems are now contemplating adoption of this new cancer screening practice or scaling-up existing screening services for the recommended populations. Thoughtful consideration of potential challenges and mitigating strategies is particularly important for anal cancer screening as the at-risk population who qualify for screening are not necessarily centralized in terms of where they seek care. In terms of clinical settings for offering anal cancer screening, the guidelines propose that this be done by primary care providers or “other specialists who interface with at-risk populations”.^7^ Patients eligible for anal cancer screening may be seen in primary care as well as in specialist HIV, transplant, or gynecologic oncology clinics, each with different workflows. Decisions regarding which facilities or clinical roles take on screening is, in and of itself, an important implementation question. Current anal cancer screening practice is widely variable among the recommended populations, ranging from non-existent to routine in a minority of care settings in high-income countries.^15-17^

With anal cancer screening, we have the opportunity to systematically plan for its adoption and implementation with thoughtful application of the concepts and methods of implementation science.^18^ Our aim is to illustrate the potential value of combining the methods of implementation science with the anal screening cascade framework to support evidence-informed adoption of the guidelines across the different clinical services points where patients might undergo anal cancer screening. Rather than dictate an idealized, one-size-fits-all plan for implementation, we hope to encourage implementers to plan strategically for local adaptation of anal cancer screening guidelines in ways that reflect the context of their settings.

### Anal Cancer Screening Cascade of Care

To support the translation of evidence into practice, we propose adoption of a cascade of care framework for anal cancer screening. The concept of care “cascades” originated from operational attempts to improve the overall effectiveness of public health efforts to address tuberculosis and sexually transmitted and blood-borne infections, including HIV and hepatitis C.^19^ Care cascades offer a structured lens for quantifying gaps in service delivery and targeting interventions where they are most needed. The underlying idea is to make explicit a sequential series of steps that typically involve identifying individuals recommended for screening, conducting a screening test, confirming whether true disease is present, treating confirmed disease, and finally retaining those treated in follow-up care and management for some duration of time. Sequential steps are depicted using a bar graph, with each bar representing the number or proportion of people who completed a step. This quantitative visualization allows for identification of important losses that can guide efforts to maximize engagement in care.

**Figure 1.**
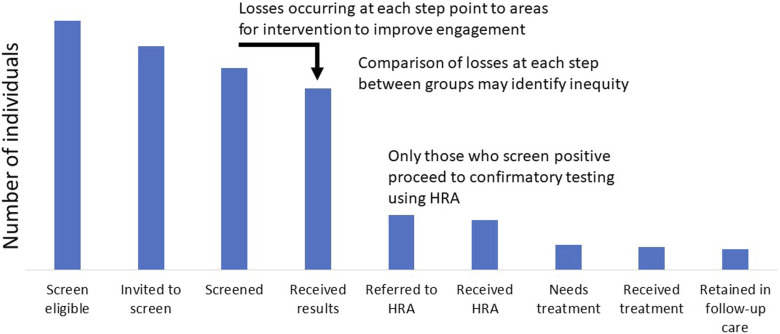
Anal cancer screening cascade of care Hypothetical and idealized depiction assuming 30% receiving an anal screening test result requiring referral to high-resolution anoscopy (HRA), 50% of those attending HRA needing treatment, and 10% losses at all other steps

Here, we apply the cascade framework to the cancer screening continuum and propose it as a starting point for consideration by implementers. In Figure 1, we present a hypothetical anal screening cascade where the denominator is all those who are eligible for screening. Along the horizontal axis, we depict the key steps along the screening continuum, starting with the total proportion of people who are screen-eligible (100%), followed by the proportion offered an initial screening test, the proportion who undergo screening, and so on through all steps of the cancer screening continuum, ending with follow-up of treated biopsy-proven (histologic) HSIL. This model of the anal screening cascade may be adapted by expanding or contracting the various steps in the care continuum as deemed relevant by its local application and data availability. For example, implementers in primary care settings may be more focused on the earlier steps to quantify the offering and uptake of screening tests and procedures,^20^ whereas those working in anal dysplasia clinics may quantify proportions of patients who have confirmed disease and return for treatment and follow-up.^21^ Although not depicted in Figure 1, implementers may also wish to examine proportions returning for timely screening following a normal screening test.^22^

For explanatory purposes, the proportions shown in Figure 1 are idealized, and may differ considerably in real-life applications. Figure 1 assumes that, along most steps, small but non-trivial losses occur (10% loss per step). In actual applications, losses may be substantially greater.^20,21,23^ For the step from the screening test to referral for confirmatory evaluation with HRA, we assumed that the proportion with positive anal screening tests would be 30% of those who undergo screening; in practice, the proportion with an abnormality may be much higher.^24^ We also assumed that only half of those who undergo HRA would require treatment.

Using actual data, the cascade approach can identify the weak links where greater losses occur to direct intensified efforts. Cardenas and colleagues applied a cascade framework using medical chart data from an HIV clinic in the United States to estimate the proportions of screen-eligible individuals who were “engaged” (having an anal Pap test) and “retained” (having a follow-up anal Pap or HRA, as indicated).^20^ Among all participants who underwent screening, they observed that 48% had an abnormal result, and, of these, 66% were retained in care, suggesting that effort was needed to encourage engagement in the earlier steps of the cascade.^20^ Similarly, Harfoush and colleagues quantified the anal cancer screening cascade among transgender women participating in a natural history study at a community clinic in the United States, with HRA information obtained via linkage with referral clinicians.^23^ Among those who underwent anal cytology, they observed that 48% had an abnormal result; of these, 89% were referred to HRA but only 42% completed it, suggesting needs for improving attendance at later steps of the cascade.^23^ These gaps are perhaps not surprising as they also occur for cervical cancer screening, such as in Guatemala, where Garcia and colleagues identified two critical “drop-off points”: (1) when individuals eligible for screening were not screened or (2) when individuals requiring confirmatory testing following abnormal screening results did not return for care.^25^ Importantly, considering the screening continuum provides a foundation upon which to guide measurement efforts, such as those done for the transition from cytology to HPV testing for cervical screening, and how that impacted colposcopy referrals.^26^

Comparisons of the proportions within individual subpopulations achieving a step between categories can identify inequities, including sex and gender disparities. To illustrate these concepts further, we share more examples from populations of people living with HIV and their clinicians, a population recommended for anal cancer screening.^24^ In a multi-site clinical HIV cohort in Ontario, Canada, we asked participants if they had undergone anal cytology testing in the past 12 months at interviews conducted in 2023, the year prior to the release of the IANS guidelines.^27^ Findings revealed that 27% of MSM aged 35 and older reported having had an anal cytology test, whereas only 5% of women and 4% of heterosexual men aged 45 and older did so.^27^ This suggests that among these guideline-recommended groups for anal screening in this setting, implementation efforts for MSM will fall more within the realm of scaling-up initiatives, whereas for the majority of women and heterosexual men living with HIV, it will be an entirely new practice and targeted efforts are needed to bring screening activities to levels experienced for MSM.

Another example from later stages of the cascade was reported by Silvera and colleagues, who followed outcomes among 1179 people diagnosed with HSIL from 2009 to 2019 in their anal dysplasia program in New York City.^21^ Although all were offered treatment, only 58% returned for treatment and of those treated, only 25% returned for follow-up HRA within 18 months. Concerningly due to high rates of recurrence (∼50%), of all with biopsy-proven HSIL, only 15% had treatment within 6 months *and* follow-up within 18 months. Those less likely to do so were people who were Black or had non-suppressed HIV viral load. These findings underline the need to investigate and address personal and structural barriers to engagement along all steps of the anal screening cascade, from start to finish. Borrowing from lessons learnt in cervical cancer surveillance, nurse navigators could provide support to decrease no show rates; in one study, no-show rates was decreased from 50% to 30%.^26^

### Implementation Science to Target Gaps Along the Anal Cancer Screening Cascade of Care

Once the anal screening cascade is enumerated, and losses are identified, the next task is to gain understanding of the reasons for the losses to enable selection of strategies to address them. Identifying a gap, in and of itself, does not provide the explanation for its occurrence. Such explanations may be revealed using the methods of implementation science that explores what works in what contexts, and why.^28^ In practice, implementation science methods apply theory and frameworks to delineate the nature of the required change, not only at the patient level but also at the provider, organization, and policy levels of health systems.^28^ For anal cancer screening, Harfoush and colleagues used qualitative inquiry to explore barriers to attending HRA among transgender women following their observance of a notable gap, and learned that socioeconomic barriers would be important to overcome.^23^ Moreover, there are many examples of the use of implementation science approaches to support better screening delivery for other cancers including colorectal, lung, breast, and cervical.^29-32^

**Figure 2.**
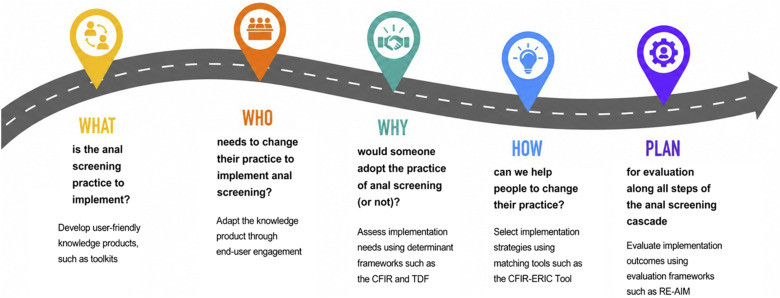
A roadmap for systematic planning for implementation of anal cancer screening, selection of implementation strategies, and evaluation^33,34^ Image generated with ChatGPT (Version 5.5), OpenAI, May 22, 2026. CFIR, consolidated framework for implementation research. TDF, theoretical domains framework. CFIR-ERIC, consolidated framework for implementation research-expert recommendations on implementing change. RE-AIM, reach effectiveness adoption implementation and maintenance

For anal cancer screening, we illustrate a potential roadmap for systematic planning, selection of implementation strategies, and evaluation (Figure 2). Implementation mapping^33^ and workbooks^34^ can help operationalize the process. Along each step of the cascade, we may consider what is the specific practice to implement, who needs to do it, and why, followed by how they could be helped to change practice, and evaluation of those change efforts. Accompanying research activities range from initial needs assessments, selection and implementation of theory-informed intervention strategies that address local barriers and facilitators, to evaluation of implementation outcomes such as the acceptability, reach, adoption, appropriateness, feasibility, cost, and sustainability of each stage along the anal screening cascade. Ideally, these activities would be done in partnership with members of the populations targeted for screening and their clinicians.

In thinking first about the “what” and “who” of the anal screening cascade, we note that it naturally divides between delivery of the anal screening test(s) and specialist HRA and treatment care services for those with an abnormal screening test result. The linkage between them is a critical step that is particularly worthy of our focus. Indeed, guideline statements urge against anal precancer screening implementation without an established clinical pathway in place for referrals to HRA. In anal cancer screening, we expect that availability of HRA would be a potential bottleneck and the triage of screening positive could overwhelm resources. In planning, this might potentially guide a stepwise approach to rollout in terms of who to offer screening first and gradually introduce the program to more individuals depending on local resources. Internationally as of 2023, it is estimated that as many as 57% of people living with HIV would be eligible for age-appropriate anal cancer screening, but that only 23% receive care at a clinic site that provides anal cytology screening, and only 16% receive care at a facility with on-site HRA services; no low-income country settings had available HRA for people living with HIV.^15^ In the United States, 33% of people living with HIV received care at a facility not known to have access to HRA services either on-site or via referral; these facilities tended to be those with low HIV caseloads that did not receive federal funding support.^16^ Without access to HRA, it would be inappropriate to offer anal precancer screening, although early-stage anal cancer screening with DARE would, however, be recommended.^7^ There is widespread agreement that it will be critical to support implementation of anal screening by improving health system capacity with greater availability of trained high-resolution anoscopists.^7^

Identifying barriers to and facilitators of anal screening implementation and applying theories and frameworks can point to implementation strategies and adaptations to explore in a given setting.^28,33^ Prior to the release of anal cancer screening recommendations, in Canada, Australia, the United States, and Nigeria, barriers and facilitators had been investigated among clinicians, mostly among those providing HIV care.^35-37^ Common themes included heath system needs for resources, importantly the scarcity of HRA and treatment specialists, referral pathways for them, and adequate financial remuneration for HRA; medical education (e.g., how to interpret screen test results) and skills-building (e.g., anal swab collection, how to communicate results to patients); clarification regarding whose clinical role it would be to provide the various steps along the cascade; and anticipation of negative reactions from patients. It will be important to learn whether these barriers and facilitators have remained the same or are different now that guideline statements are available. There may also be distinct challenges in specific populations, or for clinicians providing care to other high-risk populations such as women with a history of vulvar disease or individuals who received a solid organ transplant.

Among the populations now targeted for anal screening, exploration of barriers and facilitators has also been done prior to the emergence of the latest guidelines, primarily among people living with HIV in settings such as Canada, the United States, and Puerto Rico.^38-43^ Limited knowledge of HPV and anal cancer, and not believing that one is personally at risk is a common theme, suggesting that patient and community education will be important. Social influences are another area for intervention, most notably a recommendation from one’s doctor as a facilitator, or stigma and shame regarding anal health as a barrier to overcome. Clinician education for trauma-informed care is also likely to be important given a repeated theme that many individuals who would be offered screening have experienced sexual trauma and violence. As above, it will be important to continue to monitor local and contextual barriers and facilitators among people targeted for screening, adopt strategies to overcome them, and evaluate efforts as anal screening is scaled up and expanded to more high-risk populations beyond those living with HIV. This will be important to study at each step in the cascade, as what works for uptake of screening tests may differ from what is needed to retain individuals in treatment and follow-up care.

### Limitations of the Anal Cancer Screening Cascade Approach

We acknowledge that the cascade approach excludes some important implementation questions. As we have presented it, the endpoint for our proposed model of the anal screening cascade is follow-up care and management. That is, the cascade does not reflect what we are truly trying to prevent with screening—incidence of and mortality from anal cancer. Similarly, the model does not incorporate the return to the screen-eligible population for screen test negatives or those who have no disease upon confirmatory testing, although it could be adapted for this. It also does not address questions regarding how long management of treated HSIL should last versus returning one to standard care, or the age at which to stop screening. Similar to other healthcare cascade/continuum models,^19^ our proposed model does not depict the dynamic and longitudinal nature of the various steps within populations and over time (i.e., the concept of “churn”), nor the timeliness of each step, such as screening intervals. Nevertheless, we believe that the anal screening cascade concept has the potential to assist early implementation efforts as settings move forward with offering screening, confirmatory testing, treatment, and follow-up.

## Conclusions

We recommend the combined approaches of implementation science with the anal screening cascade framework to assist implementers with the systematic planning of data gathering to support evidence-informed implementation along all steps of the screening continuum. Applying implementation science methods can help to target efforts on greatest gaps along the cascade by considering the people, behaviors, processes, and systems needed for change and selecting strategies that overcome local barriers. As most settings are in early phases of implementation, we suggest that a needs assessment would be a good place to start, accounting for local context including which processes are already in place, which are needed, roles and capacity of clinicians, and which patients the program aims to serve. Plans to evaluate and sustain practice will benefit from attention to measures of health equity. Documentation of existing clinician needs and practices, with evaluation of early implementation efforts, can help clinicians achieve competence in screening and promote organizational change to monitor equity and reach with quality improvement initiatives, so that anal cancer screening can become part of routine care for high-risk populations. We also suggest that the combined use of a cascade framework with the methods of implementation science could be extended to care continua for other screen-preventable cancers and conditions.

## Data Availability

Not applicable. No new data were generated or analyzed for the manuscript.*
